# Event and Comment

**Published:** 1910-07-15

**Authors:** 


DENTAL REGISTER.

Vol. LXIV. July 15, 1910. No. 7.

### EVENT AND COMMENT.

We printed last month two excellent statements of the present thinking of two eminent leaders in educational work on the education of the physician. At the risk of surfeiting our readers we are printing in this issue three papers read before the American Medical Association, at St. Louis, in the section on Stomatology, last June, on the education of the medical dentist, or "Stomatologist." These papers and the discussions perhaps give us as good an idea of the extreme view of this subject as we are likely to encounter. It is to be regretted that some of our strong college men, who are actively engaged in college work, could not have been present to present the more conservative views of this subject, as we are convinced that however desirable the ends designed in this symposium, the means of attainment are not yet apparent.

The objection of these papers and discussions seems to have been the merging of the dental and medical professions, and the creation of a kind of hybrid called a stomatologist. It does not seem clear that the dentist is to become extinct, but he is of so little consequence that he must be relegated to an inferior rank, just what, is not evident. A stomatologist is defined in several instances as one who treats diseases of the mouth, and the statement is often made that he won't amount to much unless he is completely and medically educated, especially the latter. We don't belive any one will attempt to deny that a baccalaureate or even a doctorate degree would be a fine starting point for a medical or dental education, nor that the medically educated man on such a basis would not be in splendid form to take up the work of preparing himself to care for the diseases which impair mastication. But we very much question whether the time is now ready for the adoption of such requirements for the education of even stomatologists. It certainly would be a great boon to suffering humanity if all diseases of the oral cavity could be even diagnosed by the dentist and prevented when practicable. But actual conditions must be overcome before we can hope to deal with the idealistic. Do we ever stop to think that not more than ten per cent of our population has anything approaching adequate dental service, and that at the same time statistics show that ninety per cent of our people need more or less dental services, some so badly as to be in real suffering conditions of health most of the time? That is a long sentence, but it contains the practical basis on which dental educational standards will be permanently and normally settled. Ninety per cent of the members of the profession believe our profession is overcrowded, when the truth is that it would require three times as many dentists as we now have to keep the people's teeth decently clean. There is no profession to-day so sadly in need of more workers as ours. If you don't believe this statement take a day off and go through your books and see how many patients you have served during the past six months and then determine if you can the number of these whose teeth are in reasonably good condition for performing their function in a satisfactory manner. As a rule we predict it will be found that not five per cent of our patients mouths are in perfect condition, if we exclude those wearing full artificial dentures, which may or may not be in as good condition as we could hope or make them. Now the bearing of all this is that, we ought of course to improve our educational standards, but we must not loose sight of our duty. It might of course relieve matters somewhat if we could convert some of our surplus medical product into stomatologists, and in this way bring some relief to the overburdened dentists, as they could take over all surgical and much of the troublesome therapeutic work of the mouth, and give us a chance to get our patients teeth in decent repair so that the great ideal of preventive dentistry might seem a reasonable possibility in the near future. To us it seems impracticable to make medical dentists. The two lines of work while fundamentally alike are as distinct practically as medical science and the art of medical practice. A dentist might have a medical education but he is no more a physician than the man who sells life insurance with a theological education can be called a clergyman. It is a psychological if not a physical inconsistency.

It does seem as though the rational method of solving all other business propositions will have to obtain in this matter. We must recognize the fact that medicine and dentistry from the practical aspect have always been widely different callings, and these lines seem to be diverging as time goes on. The scientific department of medicine has much in common with the scientific aspect of dentistry and here there will always be a close and harmonious relationship. These standards have never been idealistic but are the result of patient and protracted research, in which dentists have done their full share. But when we come to harmonizing or unifying dental and medical practice we are certain to meet trouble and probable defeat. To practice dentistry we do not need a complete medical education, any more than we need an education in pharmacy. Now our notion of this whole matter is that our ideals of the needs of dentistry are capable of larger development, and we shall see in the next five years a tremendous realization of the great possibilities of dentistry as a preventive treatment for many ills of humanity. The demand is to be for more dentists and fewer physicians. These dentists of course must be better trained than now, and they should have different ideals than too generally prevail at present. As we view the situation our profession in the next five years will find itself wholly incapable of taking care of the work that will be demanded by an enlightened people. The sensible thing for us to do to-day is to stop fussing over the matter of our relations to other professions and the question of our rank, and get busy in endowing dental schools with buildings and men capable of training men to cope with the ever broadening field of dental needs. We must have fully equipped facilities, more time, and better teachers to train a grand army of high grade dentists to cope with the problem which is already here, and with which we are only playing. We can't wait six or eight years for our well trained medical specialists. We must use what we have, but strike for a higher development along our own lines.

If medical instructors will not do their duty by us and give our students as competent instruction, or hold our students up to as high standards, as they do the medical students we shall be forced to train up our own men to medical science teachers and so control our standards. A medical science instructor in any university or medical school who does not train his dental men completely is not worthy of his place and should be displaced by a man of broad and liberal conceptions.

We haven't space here to argue this entire question to a conclusion, but to us it needs no elaborate argument for any one who has kept in touch with the history and development of the science and art of dentistry We can't conceive of dentistry as a department of medicine in any sense other than from its scientific relationship and its general object of relieving suffering humanity. Medicine is drifting into preventive medicine as fast as its science progresses, dentistry will do the same, and it looks very much as though medicine will find its first and greatest helper in dentistry. Let us then not falter in our progress or throw away our birthright and great opportunity; but put all possible effort into the development of the scientific side of our work so that in time medical science and dental science will run along in parallel if not in confluent lines. Then the art and practice of both will have a common bond which will end what now seems like an interminable controversy merely for name and place.

